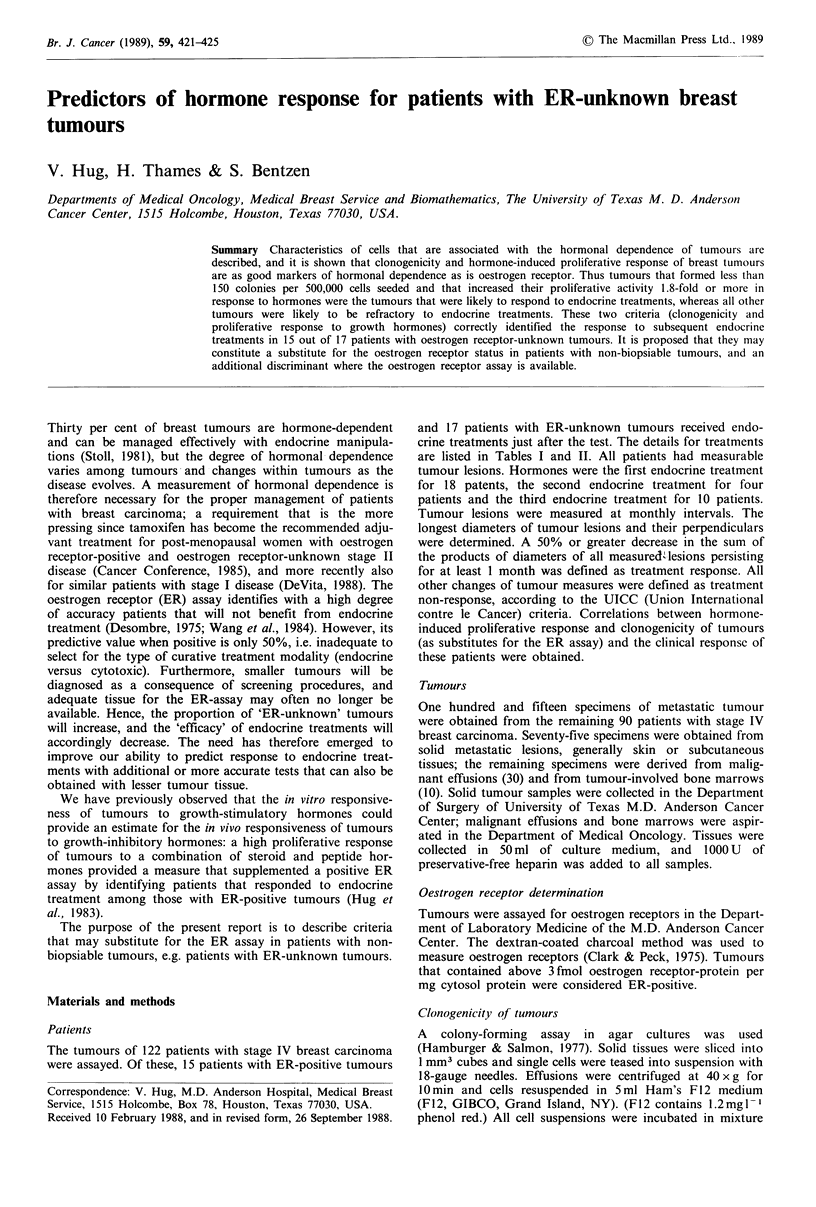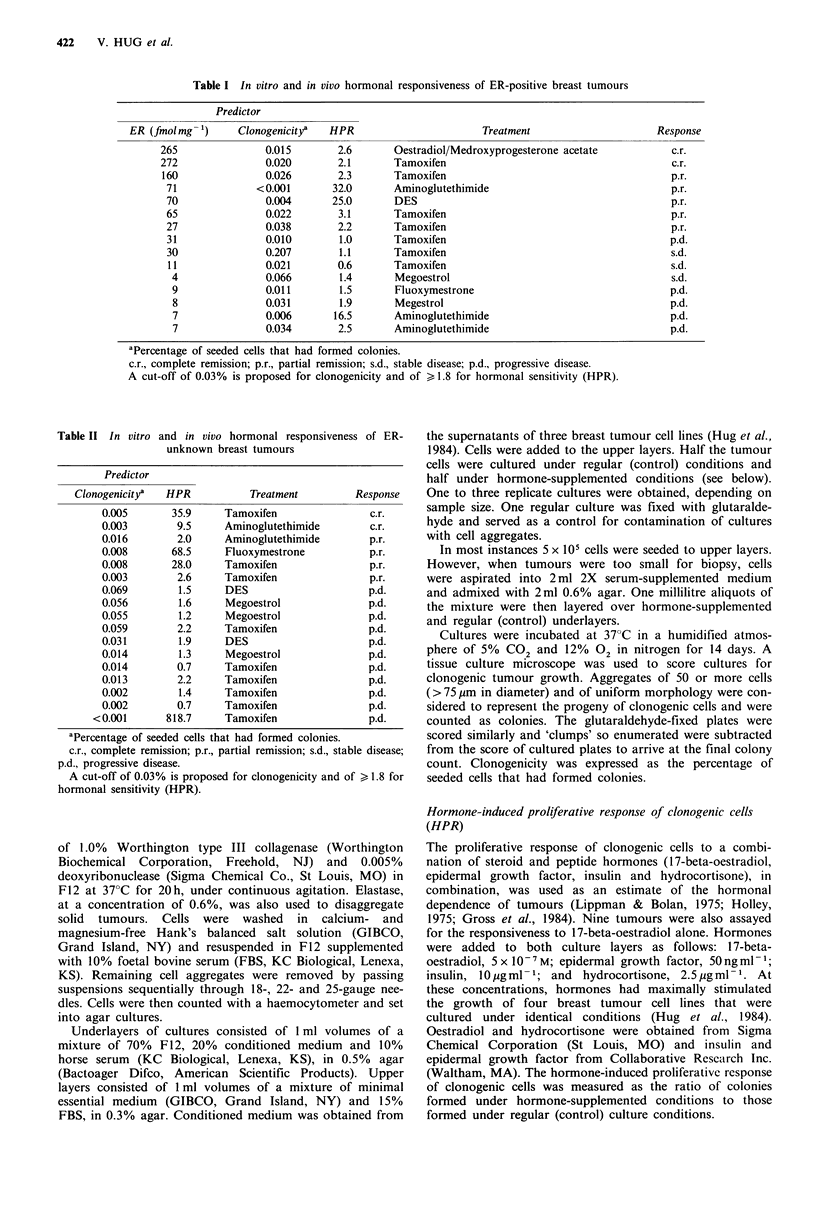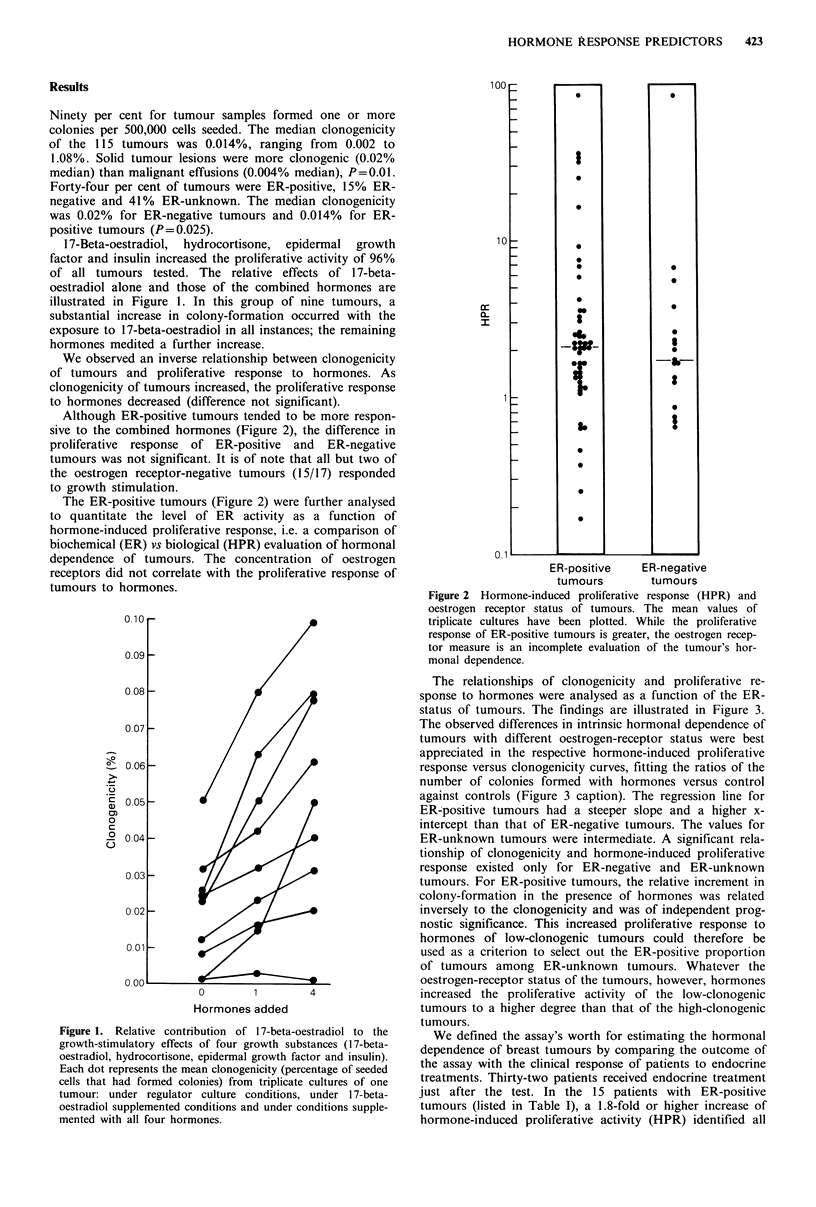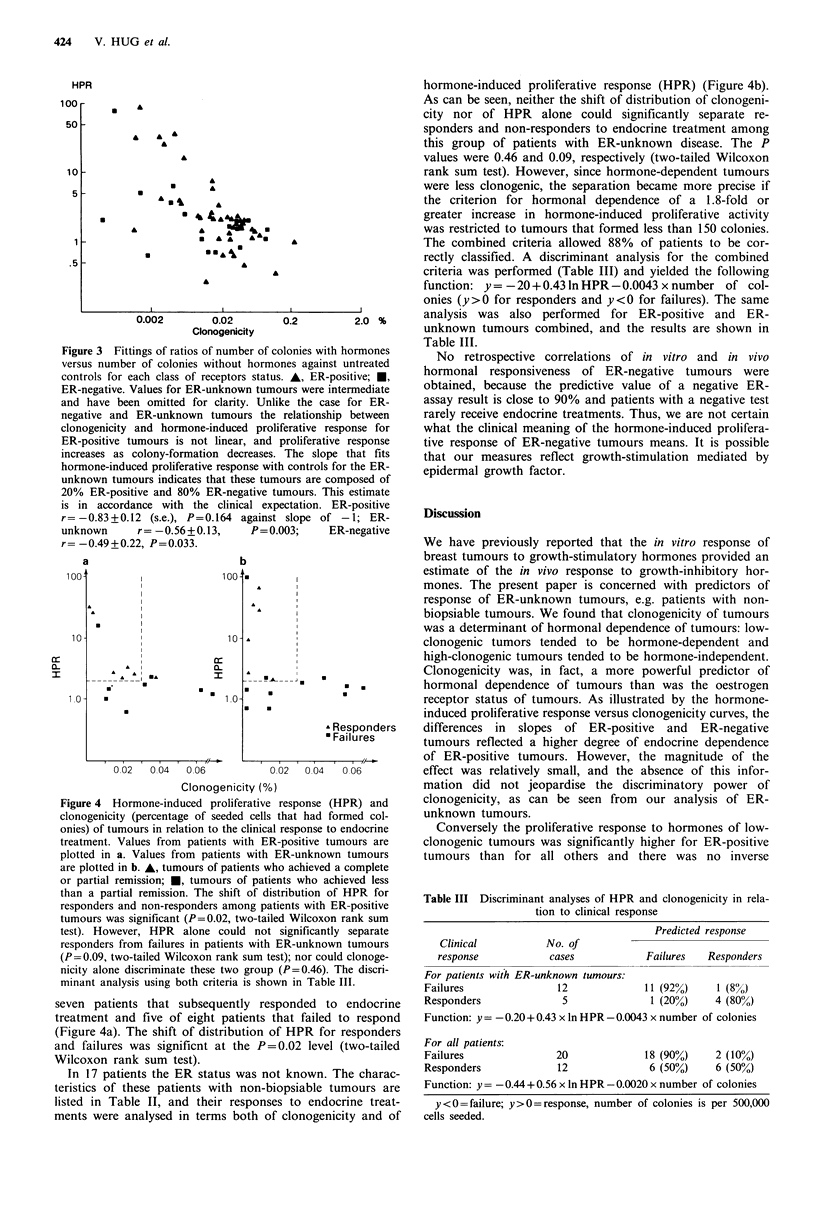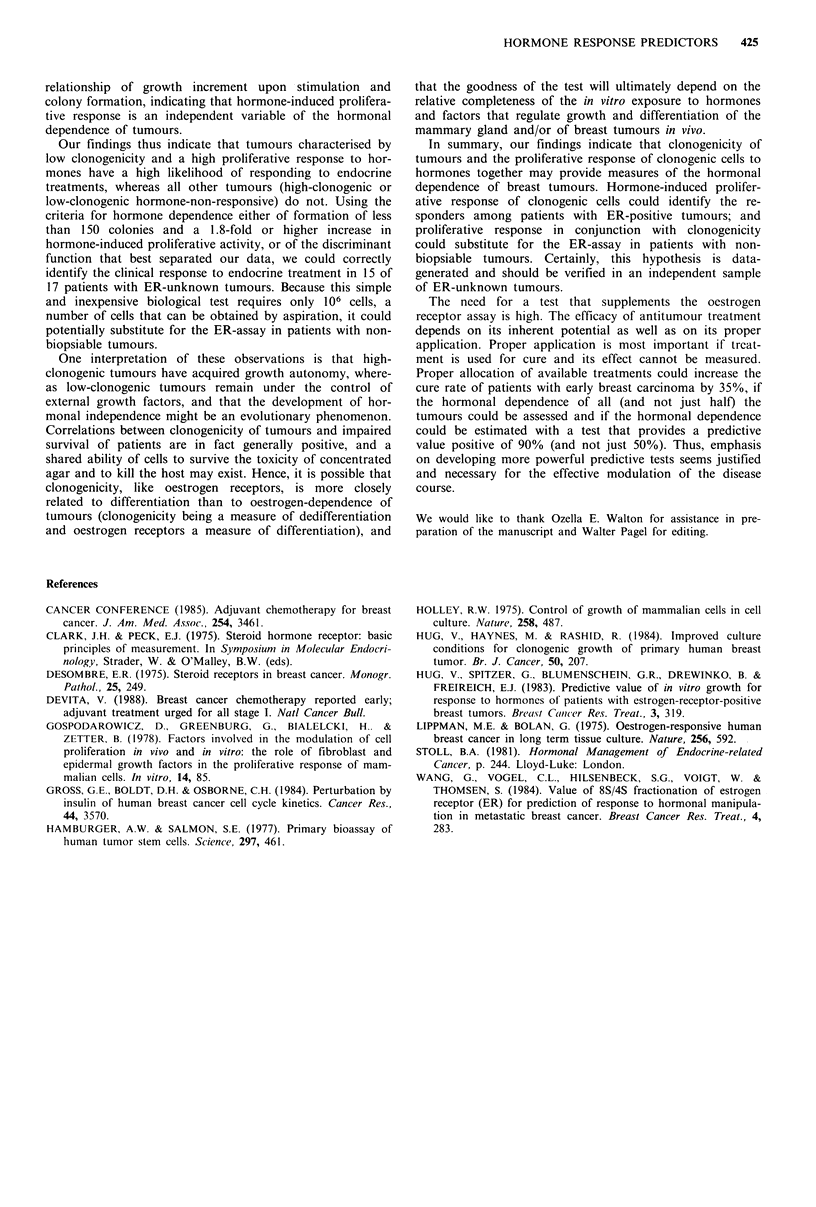# Predictors of hormone response for patients with ER-unknown breast tumours.

**DOI:** 10.1038/bjc.1989.85

**Published:** 1989-03

**Authors:** V. Hug, H. Thames, S. Bentzen

**Affiliations:** Department of Medical Oncology, University of Texas M. D. Anderson Cancer Center, Houston 77030.

## Abstract

Characteristics of cells that are associated with the hormonal dependence of tumours are described, and it is shown that clonogenicity and hormone-induced proliferative response of breast tumours are as good markers of hormonal dependence as is oestrogen receptor. Thus tumours that formed less than 150 colonies per 500,000 cells seeded and that increased their proliferative activity 1.8-fold or more in response to hormones were the tumours that were likely to respond to endocrine treatments, whereas all other tumours were likely to be refractory to endocrine treatments. These two criteria (clonogenicity and proliferative response to growth hormones) correctly identified the response to subsequent endocrine treatments in 15 out of 17 patients with oestrogen receptor-unknown tumours. It is proposed that they may constitute a substitute for the oestrogen receptor status in patients with non-biopsiable tumours, and an additional discriminant where the oestrogen receptor assay is available.


*Br. J. Cancer* (1989), **59**, 421–425 © The Macmillan Press Ltd., 1989

### Predictors of hormone response for patients with ER-unknown breast tumours

V. Hug, H. Thames & S. Bentzen

*Departments of Medical Oncology, Medical Breast Service and Biomathematics, The University of Texas M. D. Anderson Cancer Center, 1515 Holcombe, Houston, Texas 77030, USA.*

**Summary** Characteristics of cells that are associated with the hormonal dependence of tumours are described, and it is shown that clonogenicity and hormone-induced proliferative response of breast tumours are as good markers of hormonal dependence as is oestrogen receptor. Thus tumours that formed less than 150 colonies per 500,000 cells seeded and that increased their proliferative activity 1.8-fold or more in response to hormones were the tumours that were likely to respond to endocrine treatments, whereas all other tumours were likely to be refractory to endocrine treatments. These two criteria (clonogenicity and proliferative response to growth hormones) correctly identified the response to subsequent endocrine treatments in 15 out of 17 patients with oestrogen receptor-unknown tumours. It is proposed that they may constitute a substitute for the oestrogen receptor status in patients with non-biopsiable tumours, and an additional discriminant where the oestrogen receptor assay is available.

Thirty per cent of breast tumours are hormone-dependent and can be managed effectively with endocrine manipulations (Stoll, 1981), but the degree of hormonal dependence varies among tumours and changes within tumours as the disease evolves. A measurement of hormonal dependence is therefore necessary for the proper management of patients with breast carcinoma; a requirement that is the more pressing since tamoxifen has become the recommended adjuvant treatment for post-menopausal women with oestrogen receptor-positive and oestrogen receptor-unknown stage II disease (Cancer Conference, 1985), and more recently also for similar patients with stage I disease (DeVita, 1988). The oestrogen receptor (ER) assay identifies with a high degree of accuracy patients that will not benefit from endocrine treatment (Desombre, 1975; Wang *et al.*, 1984). However, its predictive value when positive is only 50%, i.e. inadequate to select for the type of curative treatment modality (endocrine versus cytotoxic). Furthermore, smaller tumours will be diagnosed as a consequence of screening procedures, and adequate tissue for the ER-assay may often no longer be available. Hence, the proportion of 'ER-unknown' tumours will increase, and the 'efficacy' of endocrine treatments will accordingly decrease. The need has therefore emerged to improve our ability to predict response to endocrine treatments with additional or more accurate tests that can also be obtained with lesser tumour tissue.

We have previously observed that the *in vitro* responsiveness of tumours to growth-stimulatory hormones could provide an estimate for the *in vivo* responsiveness of tumours to growth-inhibitory hormones: a high proliferative response of tumours to a combination of steroid and peptide hormones provided a measure that supplemented a positive ER assay by identifying patients that responded to endocrine treatment among those with ER-positive tumours (Hug *et al.*, 1983).

The purpose of the present report is to describe criteria that may substitute for the ER assay in patients with non-biopsiable tumours, e.g. patients with ER-unknown tumours.

### Materials and methods

### *Patients*

The tumours of 122 patients with stage IV breast carcinoma were assayed. Of these, 15 patients with ER-positive tumours and 17 patients with ER-unknown tumours received endocrine treatments just after the test. The details for treatments are listed in Tables I and II. All patients had measurable tumour lesions. Hormones were the first endocrine treatment for 18 patents, the second endocrine treatment for four patients and the third endocrine treatment for 10 patients. Tumour lesions were measured at monthly intervals. The longest diameters of tumour lesions and their perpendiculars were determined. A 50% or greater decrease in the sum of the products of diameters of all measured lesions persisting for at least 1 month was defined as treatment response. All other changes of tumour measures were defined as treatment non-response, according to the UICC (Union International contre le Cancer) criteria. Correlations between hormone-induced proliferative response and clonogenicity of tumours (as substitutes for the ER assay) and the clinical response of these patients were obtained.

### *Tumours*

One hundred and fifteen specimens of metastatic tumour were obtained from the remaining 90 patients with stage IV breast carcinoma. Seventy-five specimens were obtained from solid metastatic lesions, generally skin or subcutaneous tissues; the remaining specimens were derived from malignant effusions (30) and from tumour-involved bone marrows (10). Solid tumour samples were collected in the Department of Surgery of University of Texas M.D. Anderson Cancer Center; malignant effusions and bone marrows were aspirated in the Department of Medical Oncology. Tissues were collected in 50 ml of culture medium, and 1000 U of preservative-free heparin was added to all samples.

### *Oestrogen receptor determination*

Tumours were assayed for oestrogen receptors in the Department of Laboratory Medicine of the M.D. Anderson Cancer Center. The dextran-coated charcoal method was used to measure oestrogen receptors (Clark & Peck, 1975). Tumours that contained above 3 fmol oestrogen receptor-protein per mg cytosol protein were considered ER-positive.

### *Clonogenicity of tumours*

A colony-forming assay in agar cultures was used (Hamburger & Salmon, 1977). Solid tissues were sliced into 1 $mm^3$ cubes and single cells were teased into suspension with 18-gauge needles. Effusions were centrifuged at $40\times g$ for 10 min and cells resuspended in 5 ml Ham's F12 medium (F12, GIBCO, Grand Island, NY). (F12 contains 1.2 $mg\,l^{-1}$ phenol red.) All cell suspensions were incubated in mixture

Correspondence: V. Hug, M.D. Anderson Hospital, Medical Breast Service, 1515 Holcombe, Box 78, Houston, Texas 77030, USA.
Received 10 February 1988, and in revised form, 26 September 1988.

**Table I** *In vitro* and *in vivo* hormonal responsiveness of ER-positive breast tumours

| *Predictor* | | | | |
|---|---|---|---|---|
| *ER (fmol mg⁻¹)* | *Clonogenicity*[a] | *HPR* | *Treatment* | *Response* |
| 265 | 0.015 | 2.6 | Oestradiol/Medroxyprogesterone acetate | c.r. |
| 272 | 0.020 | 2.1 | Tamoxifen | c.r. |
| 160 | 0.026 | 2.3 | Tamoxifen | p.r. |
| 71 | <0.001 | 32.0 | Aminoglutethimide | p.r. |
| 70 | 0.004 | 25.0 | DES | p.r. |
| 65 | 0.022 | 3.1 | Tamoxifen | p.r. |
| 27 | 0.038 | 2.2 | Tamoxifen | p.r. |
| 31 | 0.010 | 1.0 | Tamoxifen | p.d. |
| 30 | 0.207 | 1.1 | Tamoxifen | s.d. |
| 11 | 0.021 | 0.6 | Tamoxifen | s.d. |
| 4 | 0.066 | 1.4 | Megoestrol | s.d. |
| 9 | 0.011 | 1.5 | Fluoxymestrone | p.d. |
| 8 | 0.031 | 1.9 | Megestrol | p.d. |
| 7 | 0.006 | 16.5 | Aminoglutethimide | p.d. |
| 7 | 0.034 | 2.5 | Aminoglutethimide | p.d. |

[a]Percentage of seeded cells that had formed colonies.
c.r., complete remission; p.r., partial remission; s.d., stable disease; p.d., progressive disease.
A cut-off of 0.03% is proposed for clonogenicity and of ≥1.8 for hormonal sensitivity (HPR).

**Table II** *In vitro* and *in vivo* hormonal responsiveness of ER-unknown breast tumours

| *Predictor* | | | |
|---|---|---|---|
| *Clonogenicity*[a] | *HPR* | *Treatment* | *Response* |
| 0.005 | 35.9 | Tamoxifen | c.r. |
| 0.003 | 9.5 | Aminoglutethimide | c.r. |
| 0.016 | 2.0 | Aminoglutethimide | p.r. |
| 0.008 | 68.5 | Fluoxymestrone | p.r. |
| 0.008 | 28.0 | Tamoxifen | p.r. |
| 0.003 | 2.6 | Tamoxifen | p.r. |
| 0.069 | 1.5 | DES | p.d. |
| 0.056 | 1.6 | Megoestrol | p.d. |
| 0.055 | 1.2 | Megoestrol | p.d. |
| 0.059 | 2.2 | Tamoxifen | p.d. |
| 0.031 | 1.9 | DES | p.d. |
| 0.014 | 1.3 | Megoestrol | p.d. |
| 0.014 | 0.7 | Tamoxifen | p.d. |
| 0.013 | 2.2 | Tamoxifen | p.d. |
| 0.002 | 1.4 | Tamoxifen | p.d. |
| 0.002 | 0.7 | Tamoxifen | p.d. |
| <0.001 | 818.7 | Tamoxifen | p.d. |

[a]Percentage of seeded cells that had formed colonies.
c.r., complete remission; p.r., partial remission; s.d., stable disease; p.d., progressive disease.
A cut-off of 0.03% is proposed for clonogenicity and of ≥1.8 for hormonal sensitivity (HPR).

of 1.0% Worthington type III collagenase (Worthington Biochemical Corporation, Freehold, NJ) and 0.005% deoxyribonuclease (Sigma Chemical Co., St Louis, MO) in F12 at 37°C for 20 h, under continuous agitation. Elastase, at a concentration of 0.6%, was also used to disaggregate solid tumours. Cells were washed in calcium- and magnesium-free Hank's balanced salt solution (GIBCO, Grand Island, NY) and resuspended in F12 supplemented with 10% foetal bovine serum (FBS, KC Biological, Lenexa, KS). Remaining cell aggregates were removed by passing suspensions sequentially through 18-, 22- and 25-gauge needles. Cells were then counted with a haemocytometer and set into agar cultures.

Underlayers of cultures consisted of 1 ml volumes of a mixture of 70% F12, 20% conditioned medium and 10% horse serum (KC Biological, Lenexa, KS), in 0.5% agar (Bactoager Difco, American Scientific Products). Upper layers consisted of 1 ml volumes of a mixture of minimal essential medium (GIBCO, Grand Island, NY) and 15% FBS, in 0.3% agar. Conditioned medium was obtained from the supernatants of three breast tumour cell lines (Hug *et al.*, 1984). Cells were added to the upper layers. Half the tumour cells were cultured under regular (control) conditions and half under hormone-supplemented conditions (see below). One to three replicate cultures were obtained, depending on sample size. One regular culture was fixed with glutaraldehyde and served as a control for contamination of cultures with cell aggregates.

In most instances $5 \times 10^5$ cells were seeded to upper layers. However, when tumours were too small for biopsy, cells were aspirated into 2 ml 2X serum-supplemented medium and admixed with 2 ml 0.6% agar. One millilitre aliquots of the mixture were then layered over hormone-supplemented and regular (control) underlayers.

Cultures were incubated at 37°C in a humidified atmosphere of 5% $CO_2$ and 12% $O_2$ in nitrogen for 14 days. A tissue culture microscope was used to score cultures for clonogenic tumour growth. Aggregates of 50 or more cells (>75 μm in diameter) and of uniform morphology were considered to represent the progeny of clonogenic cells and were counted as colonies. The glutaraldehyde-fixed plates were scored similarly and 'clumps' so enumerated were subtracted from the score of cultured plates to arrive at the final colony count. Clonogenicity was expressed as the percentage of seeded cells that had formed colonies.

### *Hormone-induced proliferative response of clonogenic cells (HPR)*

The proliferative response of clonogenic cells to a combination of steroid and peptide hormones (17-beta-oestradiol, epidermal growth factor, insulin and hydrocortisone), in combination, was used as an estimate of the hormonal dependence of tumours (Lippman & Bolan, 1975; Holley, 1975; Gross *et al.*, 1984). Nine tumours were also assayed for the responsiveness to 17-beta-oestradiol alone. Hormones were added to both culture layers as follows: 17-beta-oestradiol, $5 \times 10^{-7}$ M; epidermal growth factor, 50 ng ml$^{-1}$; insulin, 10 μg ml$^{-1}$; and hydrocortisone, 2.5 μg ml$^{-1}$. At these concentrations, hormones had maximally stimulated the growth of four breast tumour cell lines that were cultured under identical conditions (Hug *et al.*, 1984). Oestradiol and hydrocortisone were obtained from Sigma Chemical Corporation (St Louis, MO) and insulin and epidermal growth factor from Collaborative Research Inc. (Waltham, MA). The hormone-induced proliferative response of clonogenic cells was measured as the ratio of colonies formed under hormone-supplemented conditions to those formed under regular (control) culture conditions.

### Results

Ninety per cent for tumour samples formed one or more colonies per 500,000 cells seeded. The median clonogenicity of the 115 tumours was 0.014%, ranging from 0.002 to 1.08%. Solid tumour lesions were more clonogenic (0.02% median) than malignant effusions (0.004% median), $P=0.01$. Forty-four per cent of tumours were ER-positive, 15% ER-negative and 41% ER-unknown. The median clonogenicity was 0.02% for ER-negative tumours and 0.014% for ER-positive tumours ($P=0.025$).

17-Beta-oestradiol, hydrocortisone, epidermal growth factor and insulin increased the proliferative activity of 96% of all tumours tested. The relative effects of 17-beta-oestradiol alone and those of the combined hormones are illustrated in Figure 1. In this group of nine tumours, a substantial increase in colony-formation occurred with the exposure to 17-beta-oestradiol in all instances; the remaining hormones medited a further increase.

We observed an inverse relationship between clonogenicity of tumours and proliferative response to hormones. As clonogenicity of tumours increased, the proliferative response to hormones decreased (difference not significant).

Although ER-positive tumours tended to be more responsive to the combined hormones (Figure 2), the difference in proliferative response of ER-positive and ER-negative tumours was not significant. It is of note that all but two of the oestrogen receptor-negative tumours (15/17) responded to growth stimulation.

The ER-positive tumours (Figure 2) were further analysed to quantitate the level of ER activity as a function of hormone-induced proliferative response, i.e. a comparison of biochemical (ER) *vs* biological (HPR) evaluation of hormonal dependence of tumours. The concentration of oestrogen receptors did not correlate with the proliferative response of tumours to hormones.

**Figure 1.** Relative contribution of 17-beta-oestradiol to the growth-stimulatory effects of four growth substances (17-beta-oestradiol, hydrocortisone, epidermal growth factor and insulin). Each dot represents the mean clonogenicity (percentage of seeded cells that had formed colonies) from triplicate cultures of one tumour: under regulator culture conditions, under 17-beta-oestradiol supplemented conditions and under conditions supplemented with all four hormones.

**Figure 2** Hormone-induced proliferative response (HPR) and oestrogen receptor status of tumours. The mean values of triplicate cultures have been plotted. While the proliferative response of ER-positive tumours is greater, the oestrogen receptor measure is an incomplete evaluation of the tumour's hormonal dependence.

The relationships of clonogenicity and proliferative response to hormones were analysed as a function of the ER-status of tumours. The findings are illustrated in Figure 3. The observed differences in intrinsic hormonal dependence of tumours with different oestrogen-receptor status were best appreciated in the respective hormone-induced proliferative response versus clonogenicity curves, fitting the ratios of the number of colonies formed with hormones versus control against controls (Figure 3 caption). The regression line for ER-positive tumours had a steeper slope and a higher x-intercept than that of ER-negative tumours. The values for ER-unknown tumours were intermediate. A significant relationship of clonogenicity and hormone-induced proliferative response existed only for ER-negative and ER-unknown tumours. For ER-positive tumours, the relative increment in colony-formation in the presence of hormones was related inversely to the clonogenicity and was of independent prognostic significance. This increased proliferative response to hormones of low-clonogenic tumours could therefore be used as a criterion to select out the ER-positive proportion of tumours among ER-unknown tumours. Whatever the oestrogen-receptor status of the tumours, however, hormones increased the proliferative activity of the low-clonogenic tumours to a higher degree than that of the high-clonogenic tumours.

We defined the assay's worth for estimating the hormonal dependence of breast tumours by comparing the outcome of the assay with the clinical response of patients to endocrine treatments. Thirty-two patients received endocrine treatment just after the test. In the 15 patients with ER-positive tumours (listed in Table I), a 1.8-fold or higher increase of hormone-induced proliferative activity (HPR) identified all

**Figure 3** Fittings of ratios of number of colonies with hormones versus number of colonies without hormones against untreated controls for each class of receptors status. ▲, ER-positive; ■, ER-negative. Values for ER-unknown tumours were intermediate and have been omitted for clarity. Unlike the case for ER-negative and ER-unknown tumours the relationship between clonogenicity and hormone-induced proliferative response for ER-positive tumours is not linear, and proliferative response increases as colony-formation decreases. The slope that fits hormone-induced proliferative response with controls for the ER-unknown tumours indicates that these tumours are composed of 20% ER-positive and 80% ER-negative tumours. This estimate is in accordance with the clinical expectation. ER-positive $r=-0.83\pm0.12$ (s.e.), $P=0.164$ against slope of $-1$; ER-unknown $r=-0.56\pm0.13$, $P=0.003$; ER-negative $r=-0.49\pm0.22$, $P=0.033$.

**Figure 4** Hormone-induced proliferative response (HPR) and clonogenicity (percentage of seeded cells that had formed colonies) of tumours in relation to the clinical response to endocrine treatment. Values from patients with ER-positive tumours are plotted in **a**. Values from patients with ER-unknown tumours are plotted in **b**. ▲, tumours of patients who achieved a complete or partial remission; ■, tumours of patients who achieved less than a partial remission. The shift of distribution of HPR for responders and non-responders among patients with ER-positive tumours was significant ($P=0.02$, two-tailed Wilcoxon rank sum test). However, HPR alone could not significantly separate responders from failures in patients with ER-unknown tumours ($P=0.09$, two-tailed Wilcoxon rank sum test); nor could clonogenicity alone discriminate these two group ($P=0.46$). The discriminant analysis using both criteria is shown in Table III.

seven patients that subsequently responded to endocrine treatment and five of eight patients that failed to respond (Figure 4a). The shift of distribution of HPR for responders and failures was significnt at the $P=0.02$ level (two-tailed Wilcoxon rank sum test).

In 17 patients the ER status was not known. The characteristics of these patients with non-biopsiable tumours are listed in Table II, and their responses to endocrine treatments were analysed in terms both of clonogenicity and of hormone-induced proliferative response (HPR) (Figure 4b). As can be seen, neither the shift of distribution of clonogenicity nor of HPR alone could significantly separate responders and non-responders to endocrine treatment among this group of patients with ER-unknown disease. The $P$ values were 0.46 and 0.09, respectively (two-tailed Wilcoxon rank sum test). However, since hormone-dependent tumours were less clonogenic, the separation became more precise if the criterion for hormonal dependence of a 1.8-fold or greater increase in hormone-induced proliferative activity was restricted to tumours that formed less than 150 colonies. The combined criteria allowed 88% of patients to be correctly classified. A discriminant analysis for the combined criteria was performed (Table III) and yielded the following function: $y=-20+0.43\ln\text{HPR}-0.0043\times\text{number}$ of colonies ($y>0$ for responders and $y<0$ for failures). The same analysis was also performed for ER-positive and ER-unknown tumours combined, and the results are shown in Table III.

No retrospective correlations of *in vitro* and *in vivo* hormonal responsiveness of ER-negative tumours were obtained, because the predictive value of a negative ER-assay result is close to 90% and patients with a negative test rarely receive endocrine treatments. Thus, we are not certain what the clinical meaning of the hormone-induced proliferative response of ER-negative tumours means. It is possible that our measures reflect growth-stimulation mediated by epidermal growth factor.

### Discussion

We have previously reported that the *in vitro* response of breast tumours to growth-stimulatory hormones provided an estimate of the *in vivo* response to growth-inhibitory hormones. The present paper is concerned with predictors of response of ER-unknown tumours, e.g. patients with non-biopsiable tumours. We found that clonogenicity of tumours was a determinant of hormonal dependence of tumours: low-clonogenic tumors tended to be hormone-dependent and high-clonogenic tumours tended to be hormone-independent. Clonogenicity was, in fact, a more powerful predictor of hormonal dependence of tumours than was the oestrogen receptor status of tumours. As illustrated by the hormone-induced proliferative response versus clonogenicity curves, the differences in slopes of ER-positive and ER-negative tumours reflected a higher degree of endocrine dependence of ER-positive tumours. However, the magnitude of the effect was relatively small, and the absence of this information did not jeopardise the discriminatory power of clonogenicity, as can be seen from our analysis of ER-unknown tumours.

Conversely the proliferative response to hormones of low-clonogenic tumours was significantly higher for ER-positive tumours than for all others and there was no inverse

**Table III** Discriminant analyses of HPR and clonogenicity in relation to clinical response

| Clinical response | No. of cases | Predicted response: Failures | Predicted response: Responders |
|---|---|---|---|
| *For patients with ER-unknown tumours:* | | | |
| Failures | 12 | 11 (92%) | 1 (8%) |
| Responders | 5 | 1 (20%) | 4 (80%) |
| Function: $y=-0.20+0.43\times\ln\text{HPR}-0.0043\times\text{number of colonies}$ | | | |
| *For all patients:* | | | |
| Failures | 20 | 18 (90%) | 2 (10%) |
| Responders | 12 | 6 (50%) | 6 (50%) |
| Function: $y=-0.44+0.56\times\ln\text{HPR}-0.0020\times\text{number of colonies}$ | | | |

$y<0$ = failure; $y>0$ = response, number of colonies is per 500,000 cells seeded.

relationship of growth increment upon stimulation and colony formation, indicating that hormone-induced proliferative response is an independent variable of the hormonal dependence of tumours.

Our findings thus indicate that tumours characterised by low clonogenicity and a high proliferative response to hormones have a high likelihood of responding to endocrine treatments, whereas all other tumours (high-clonogenic or low-clonogenic hormone-non-responsive) do not. Using the criteria for hormone dependence either of formation of less than 150 colonies and a 1.8-fold or higher increase in hormone-induced proliferative activity, or of the discriminant function that best separated our data, we could correctly identify the clinical response to endocrine treatment in 15 of 17 patients with ER-unknown tumours. Because this simple and inexpensive biological test requires only $10^6$ cells, a number of cells that can be obtained by aspiration, it could potentially substitute for the ER-assay in patients with non-biopsiable tumours.

One interpretation of these observations is that high-clonogenic tumours have acquired growth autonomy, whereas low-clonogenic tumours remain under the control of external growth factors, and that the development of hormonal independence might be an evolutionary phenomenon. Correlations between clonogenicity of tumours and impaired survival of patients are in fact generally positive, and a shared ability of cells to survive the toxicity of concentrated agar and to kill the host may exist. Hence, it is possible that clonogenicity, like oestrogen receptors, is more closely related to differentiation than to oestrogen-dependence of tumours (clonogenicity being a measure of dedifferentiation and oestrogen receptors a measure of differentiation), and that the goodness of the test will ultimately depend on the relative completeness of the *in vitro* exposure to hormones and factors that regulate growth and differentiation of the mammary gland and/or of breast tumours *in vivo*.

In summary, our findings indicate that clonogenicity of tumours and the proliferative response of clonogenic cells to hormones together may provide measures of the hormonal dependence of breast tumours. Hormone-induced proliferative response of clonogenic cells could identify the responders among patients with ER-positive tumours; and proliferative response in conjunction with clonogenicity could substitute for the ER-assay in patients with non-biopsiable tumours. Certainly, this hypothesis is data-generated and should be verified in an independent sample of ER-unknown tumours.

The need for a test that supplements the oestrogen receptor assay is high. The efficacy of antitumour treatment depends on its inherent potential as well as on its proper application. Proper application is most important if treatment is used for cure and its effect cannot be measured. Proper allocation of available treatments could increase the cure rate of patients with early breast carcinoma by 35%, if the hormonal dependence of all (and not just half) the tumours could be assessed and if the hormonal dependence could be estimated with a test that provides a predictive value positive of 90% (and not just 50%). Thus, emphasis on developing more powerful predictive tests seems justified and necessary for the effective modulation of the disease course.

We would like to thank Ozella E. Walton for assistance in preparation of the manuscript and Walter Pagel for editing.

### References

CANCER CONFERENCE (1985). Adjuvant chemotherapy for breast cancer. *J. Am. Med. Assoc.*, **254**, 3461.

CLARK, J.H. & PECK, E.J. (1975). Steroid hormone receptor: basic principles of measurement. In *Symposium in Molecular Endocrinology*, Strader, W. & O'Malley, B.W. (eds).

DESOMBRE, E.R. (1975). Steroid receptors in breast cancer. *Monogr. Pathol.*, **25**, 249.

DEVITA, V. (1988). Breast cancer chemotherapy reported early; adjuvant treatment urged for all stage I. *Natl Cancer Bull.*

GOSPODAROWICZ, D., GREENBURG, G., BIALELCKI, H. & ZETTER, B. (1978). Factors involved in the modulation of cell proliferation *in vivo* and *in vitro*: the role of fibroblast and epidermal growth factors in the proliferative response of mammalian cells. *In vitro*, **14**, 85.

GROSS, G.E., BOLDT, D.H. & OSBORNE, C.H. (1984). Perturbation by insulin of human breast cancer cell cycle kinetics. *Cancer Res.*, **44**, 3570.

HAMBURGER, A.W. & SALMON, S.E. (1977). Primary bioassay of human tumor stem cells. *Science*, **297**, 461.

HOLLEY, R.W. 1975). Control of growth of mammalian cells in cell culture. *Nature*, **258**, 487.

HUG, V., HAYNES, M. & RASHID, R. (1984). Improved culture conditions for clonogenic growth of primary human breast tumor. *Br. J. Cancer*, **50**, 207.

HUG, V., SPITZER, G., BLUMENSCHEIN, G.R., DREWINKO, B. & FREIREICH, E.J. (1983). Predictive value of *in vitro* growth for response to hormones of patients with estrogen-receptor-positive breast tumors. *Breast Cancer Res. Treat.*, **3**, 319.

LIPPMAN, M.E. & BOLAN, G. (1975). Oestrogen-responsive human breast cancer in long term tissue culture. *Nature*, **256**, 592.

STOLL, B.A. (1981). *Hormonal Management of Endocrine-related Cancer*, p. 244. Lloyd-Luke: London.

WANG, G., VOGEL, C.L., HILSENBECK, S.G., VOIGT, W. & THOMSEN, S. (1984). Value of 8S/4S fractionation of estrogen receptor (ER) for prediction of response to hormonal manipulation in metastatic breast cancer. *Breast Cancer Res. Treat.*, **4**, 283.